# Editorial: Bridging gaps in understanding consciousness: multidisciplinary perspectives

**DOI:** 10.3389/fpsyg.2026.1881865

**Published:** 2026-06-29

**Authors:** Poppy Schoenberg, Daniela Domingues, Luca Ronconi, Andrea Galbiati

**Affiliations:** 1 Vanderbilt University Medical Center, Nashville, TN, United States; 2 NCIS, National Coalition of Independent Scholars, Brattleboro, VT, United States; 3 Paris Brain Institute—ICM, Sorbonne Université, Inserm, CNRS, AP-HP Hôpital de la Pitié Salpêtrière, Paris, France; 4 Department of Psychology and Cognitive Science, University of Trento, Rovereto, Italy; 5 Vita-Salute San Raffale University, Psychology, Milan, Italy; 6 IRCCS San Raffaele Scientific Institute, Clinical Neurosciences, Neurology-Sleep Disorder Center, Milan, Italy

**Keywords:** cognitive neuroscience, conscious experience, consciousness research, interdisciplinary approaches, neural correlates, theoretical frameworks

Mapping the concepts, mechanisms, and processes that give rise to conscious, preconscious, and unconscious experience remains one of the most enduring challenges in contemporary science. Philosophical reflections around consciousness are millennia old, although its systematic empirical investigation is relatively recent, wherein “consciousness science” has become its own discipline within the last three decades. Progress has been made across domains, including philosophy and physics for conceptual clarity and theoretical grounding; neuroscience and neuroimaging for the measurement of neural networks and interactions; computational modeling to formalize principles and generate testable predictions; behavioral science paradigms to quantify subjective experience; and clinical and developmental studies in which insights from patient and population cohorts inform adaptive and maladaptive operations of consciousness. Collectively, these disciplines provide powerful frameworks and tools for investigating “consciousness.” They enrich understanding of the structures and dynamics underlying diverse biological and cognitive systems, potentially extending to other life forms/systems. Albeit, integrating multi-dimensional insights across levels of analysis, rather than pursuing a single unified framework, remains a central scientific challenge.

Our intention with this Research Topic, *Bridging Gaps in Understanding Consciousness: Multidisciplinary Perspectives*, was to pursue measured steps toward addressing these challenges by bringing together contributions that illuminate the diverse approaches currently shaping contemporary consciousness science research, and advancing its multi-leveled understanding. Without leaning too strictly into semantics; hypotheses provide targeted, testable claims about specific mechanisms, whereas theories aim to integrate diverse findings into a coherent and comprehensive framework, describing broader relationships among processes, functions, and phenomena across levels of analysis. As such, we welcomed work that refined extant models and data, alongside contributions that brought new perspectives to the field.

The 12 articles, involving 33 authors, within this Research Topic bridge these (sub)specialties, highlighting cross-method and cross-disciplinary connections. Several contributions focus on theoretical foundations. In a thoughtful conceptual analysis, Marchetti provides an incisive examination of the relationship between conscious experience and self-construction, offering insights into how subjective experience shapes self-representation and awareness. They propose that the phenomenal mode of consciousness is multi-dimensional across five main dimensions that encompass the qualitative, quantitative, hedonic, temporal, and spatial. In a related theoretical contribution, Marchetti advances an attention-based account of emotion, persuasively showing how emotional experiences emerge from modulations within attentional systems that structure conscious awareness. Taken together, these works support the integration of affective and self-related processes into broader theoretical models of consciousness.

Other theoretical articles introduce novel frameworks for understanding the structure and dynamics of conscious phenomena. Páleník presents a compelling narrative review that outlines the increasingly influential perspective of consciousness as a multidimensional phenomenon, thoughtfully discussing the conceptual and methodological challenges associated with defining and measuring its dimensions. Tleuberdinova offers a bold and imaginative hypothesis regarding the origin of Earth's universal bioconsciousness, situating the emergence of conscious processes within an extended evolutionary and planetary context. Worden contributes a pioneering “Projective Wave Theory of Consciousness,” proposing that conscious experience in the mammalian brain arises from a wave-like excitation within the thalamus which encodes 3-D spatial information in an analog form akin to a hologram, that is, discrete from standard neural computation. This thesis aligns with emerging work in the philosophy of biology and comparative neuroscience that emphasizes the role of large-scale dynamical and oscillatory processes in supporting conscious experience across diverse nervous systems, operating in conjunction with, and extending beyond, purely computational accounts. Collectively, these theoretical contributions highlight the creativity, breadth, and conceptual robustness of contemporary consciousness science.

A related theme emphasizes the development of methodological frameworks capable of operationalizing consciousness. Pradhan offers a meticulous approach grounded in engineering principles to the longstanding challenge of measuring consciousness, presenting a structured pragmatic pipeline that guides researchers from modeling to sensing, interpretation, and validation. Pradhan defines the components of a “consciousness measurement system (CMS),” establishes criteria for measurability, and systematically applies them across different use cases, illustrating when current theories or technologies can(not) quantify specific aspects of conscious processes. This work demonstrates how careful methodological design can translate conceptual models into empirically testable metrics that are comprehensive and systematic, enhancing the field's capacity to generate valid reproducible outcomes.

Other contributions focus on the neural and physiological mechanisms underlying conscious processing. Bandara and Hutchinson provide an analytical perspective on the widely used “seen–unseen contrast” in consciousness research, critically noting that this approach alone cannot identify sufficient neural markers of conscious experience, and thus advocate for the development of broader experimental designs to facilitate experimental rigor. Dai et al. report a well-executed empirical investigation of network dysregulation in disorders of consciousness using functional magnetic resonance imaging (fMRI) data. They employed a novel minimum spanning tree analysis to reveal alterations in brain network organization linked to impaired awareness, particularly reflecting disruptions in global efficiency and network integration that may be detrimental to information transfer across large-scale neural systems. Di Dona and Ronconi offer an encompassing review of evidence implicating beta oscillations in visual processing as a potential mechanism supporting preconscious perception within the dorsal visual stream. Overall, these studies underscore the growing value of electrophysiology, network neuroscience, and systems-level analysis for elucidating the neural correlates of (pre-)conscious processing.

Empirical investigations also shed light on behavioral and experiential dimensions of consciousness. Kim et al. present an elegant study on mirror illusions during mirror therapy, showing how perceptual and sensorimotor factors shape the experience of body ownership and visual feedback. Beyond its clinical relevance, this work provides a translational lens into how embodied perception contributes to conscious awareness, offering insights into the mechanisms by which sensory integration and motor predictions modulate subjective experience. Massullo et al. provide a methodologically innovative evaluation of a “fact sheet” designed to communicate evidence-based explanations for reported ghostly experiences, demonstrating how empirical approaches can engage phenomena that often fall outside conventional experimental paradigms. Together, these contributions illustrate how careful experimental design can effectively bridge theoretical frameworks with observable experience.

Finally, the Research Topic includes work applying theoretical perspectives to psychological phenomena. Sun and Jin provide a detailed meta-abductive account of depression conceptualized as inferential rigidity, showing how rigid cognitive processes may shape experience and cognition that provides explanatory power for depression's cognitive persistence and resistance to intervention. Their framework proposes the integration of philosophical psychiatry and clinical theory, offering a valuable bridge between consciousness research and applied psychological science.

The goal of this Research Topic was not to resolve longstanding philosophical debates surrounding consciousness, or enter into semantics regarding terminology, but rather to stimulate dialogue and encourage cross-disciplinary exchange in connecting the extant purview. In sum, this Research Topic demonstrates the richness of contemporary consciousness science research, showing how and why disparate approaches can inform one another. By bridging disciplines, these contributions underscore the benefit of integrating insights across multiple levels of analysis, refining existing models, and remaining prudently open to novel perspectives. Currently, no single model or framework captures this scope. However, the careful combination of conceptual rigor, empirical precision, and methodological innovation exemplified here provides a map for continued progress, and over time, may contribute to the development of an increasingly expansive understanding. We hope this volume inspires further advancement of a science of consciousness that charts its remarkable depth, complexity, and nuance.

